# Potential of Global Cropland Phytolith Carbon Sink from Optimization of Cropping System and Fertilization

**DOI:** 10.1371/journal.pone.0073747

**Published:** 2013-09-16

**Authors:** Zhaoliang Song, Jeffrey F. Parr, Fengshan Guo

**Affiliations:** 1 Zhejiang Provincial Key Laboratory of C Cycling in Forest Ecosystems and C Sequestration, Zhejiang Agricultural and Forestry University, Lin’an, Zhejiang, China; 2 School of Environment and Resources, Zhejiang Agricultural and Forestry University, Lin’an, Zhejiang, China; 3 State Key Laboratory of Environmental Geochemistry, Institute of Geochemistry, Chinese Academy of Sciences, Guiyang, Guizhou, China; 4 Southern Cross GeoScience, Southern Cross University, Lismore, New South Wales, Australia; University of Connecticut, United States of America

## Abstract

The occlusion of carbon (C) by phytoliths, the recalcitrant silicified structures deposited within plant tissues, is an important persistent C sink mechanism for croplands and other grass-dominated ecosystems. By constructing a silica content-phytolith content transfer function and calculating the magnitude of phytolith C sink in global croplands with relevant crop production data, this study investigated the present and potential of phytolith C sinks in global croplands and its contribution to the cropland C balance to understand the cropland C cycle and enhance long-term C sequestration in croplands. Our results indicate that the phytolith sink annually sequesters 26.35±10.22 Tg of carbon dioxide (CO_2_) and may contribute 40±18% of the global net cropland soil C sink for 1961–2100. Rice (25%), wheat (19%) and maize (23%) are the dominant contributing crop species to this phytolith C sink. Continentally, the main contributors are Asia (49%), North America (17%) and Europe (16%). The sink has tripled since 1961, mainly due to fertilizer application and irrigation. Cropland phytolith C sinks may be further enhanced by adopting cropland management practices such as optimization of cropping system and fertilization.

## Introduction

Present understanding of the global carbon (C) cycle and climate feedbacks is limited by uncertainty over terrestrial C balance [1]–[5]. As one of the largest terrestrial ecosystems deeply influenced by human activities, the croplands cover an area of 15.33×10^8^ hm^2^ globally and may play a significant role in terrestrial C balance [3], [6]. Although croplands were traditionally considered to be the largest biospheric source of C lost to the atmosphere in most areas of the world [7]–[12], they may also be significant C sinks under proper management [3], [6], [13]–[15].

Phytolith-occluded C (PhytOC), where C is entrapped within recalcitrant silicified structures when they are deposited within plant tissues [16]–[18], is particularly prolific in many crops such as rice [19], wheat [20], millet [21] and sugarcane [22]. PhytOC is highly resistant against decomposition [18], [23]–[25] and may accumulate in soil for several thousands of years after plant decomposition [18], demonstrating the potential of phytoliths in the long-term biogeochemical sequestration of atmospheric carbon dioxide (CO_2_) [5], [26]. Soil PhytOC accumulation is an important persistent C sink mechanism for croplands [18]–[22] and other grass-dominated ecosystems [26], [27]. Moreover, Jansson et. al. [28] suggest that the production of PhytOC in croplands could be greatly enhanced through crop breeding. However, the present and potential of global cropland phytolith C sink have not been revealed.

In the present study, we quantifed the present and potential of phytolith carbon sink and its contribution to the global cropland C balance by constructing a silica content- phytolith content transfer function and calculating the magnitude of the phytolith C sink in global croplands with relevant crop data including the PhytOC and silica content, farm crop output, the Si-rich organ ratio (mass ratios of the Si-rich organ: crop output) and the PhytOC stability factor. The purposes of the study are to guide the management of cropland ecosystems to maximize phytolith C sequestration and mitigate climate change.

## Materials and Methods

### Ethics Statements

No specific permits were required for the described field studies, because the experimental field is owned by Zhejiang Agricultural and Forestry University, and the School of Environment and Resources performs the management. No specific permits were required for these locations/activities, because the location is not privately-owned or protected in any way, the field studies did not involve endangered or protected species, and each sample consisted of no more than 500 grams (fresh weight).

### Constructing the Transfer Function for the Phytolith:Silica Content

Plant phytolith content may be estimated from plant silica content data using the transfer function for the phytolith:silica content [26]. To construct the silica content- phytolith content transfer function, mature crop organ samples were collected– each sample consisted of approximately 500 g of composite plant material.

Plant samples were oven-dried at 65°C to a constant mass and cut into small pieces (<5 mm). They were ashed at 500°C to remove organic matter, fused with lithium metaborate, dissolved in dilute nitric acid and analyzed for silica content using inductively coupled plasma-optical emission spectroscopy (ICP-OES; Optima 7000 DV, Perkin Elmer, Massachusetts, USA). Plant phytoliths were isolated using a microwave digestion process followed by a Walkley–Black type digestion to ensure the removal of extraneous organic material [19], [27]. The isolated phytoliths were dried to a constant mass at 75°C for 24 h in a fan-forced oven and weighed to determine the plant phytolith content. The occluded-C content within phytoliths was also determined [19], [27]. The error was <5% in phytolith and silica measurements and <10% in PhytOC measurements using plant standards (GSV-1) and triplicate analyses.

**Figure 1:**
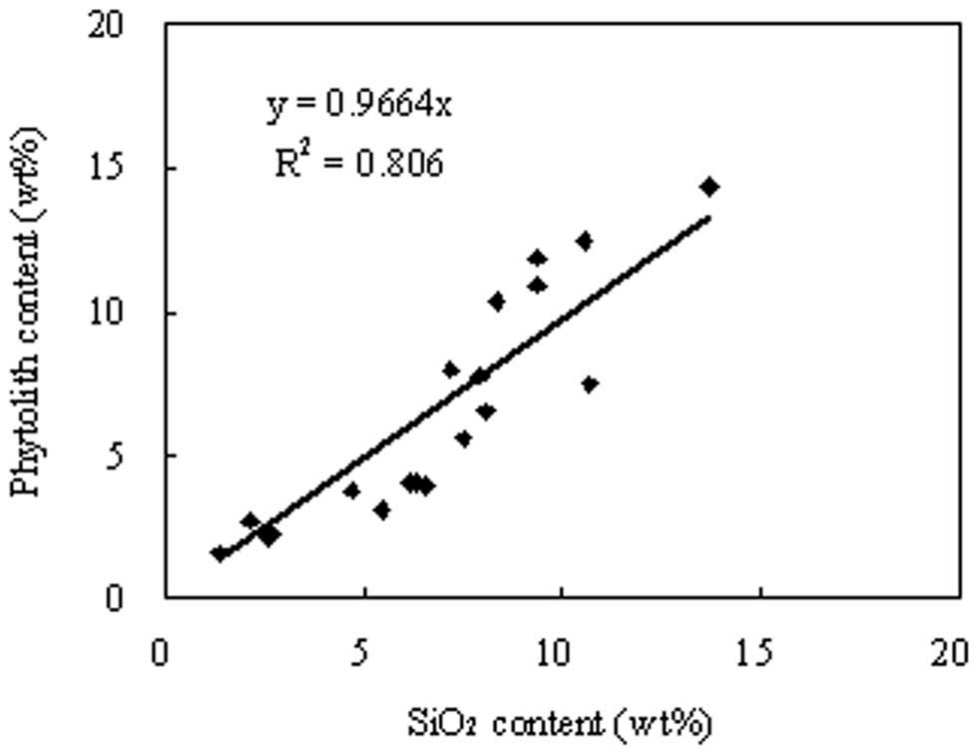
The correlation of phytolith content (%) to SiO_2_ (%) content in different crop species (p<0.01).

The plant silica content- phytolith content transfer function was constructed using regression analysis based on the phytolith and silica contents determined for the samples (Figure 1). Silica content was converted to phytolith content using the following equation (R^2^ = 0.806, p<0.01):
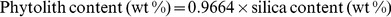
(1)


### Data Collection, Phytolith and PhytOC Content Estimation

Farm productivity data was obtained from Food and Agriculture Organization of the United Nations (FAO) Statistics [29]. Silica content data was obtained from published monographs [30], [31], papers [19]–[21], [32], [33] and also determined in the present study. Silica content of crop species was used to estimate phytolith content using a conversion factor of 0.9664; see equation (1). The PhytOC content in plant organs was estimated from phytolith content data using an occluded-C content in phytolith of 2∼4% (average 3%) according to the present study and references [19]–[21], [27].

### Estimating PhytOC Production and the Phytolith C Sink

The production of PhytOC is primarily affected by plant PhytOC concentration and aboveground net primary productivity (ANPP) of Si-rich organs [34], where plant PhytOC is mainly determined by PhytOC content in phytoliths [26] and plant Si content [30], [33]. This allowed the crop PhytOC production rate to be estimated from the PhytOC content and total ANPP of the Si-rich organs of an area as:
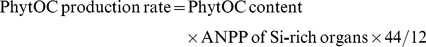
(2)where PhytOC production rate is the PhytOC production by a particular crop’s Si-rich organs per year (Tg CO_2_ yr^–1^), PhytOC content is the concentration of PhytOC in a crop’s Si-rich organs (wt %) and ANPP is the total aboveground net primary productivity of Si-rich crop organs (Tg yr^–1^) of an area estimated from Si-rich organ factor [35], [36] and crop output [29].

As the PhytOC sequestration rate is controlled by the PhytOC production rate in plants and the stability of phytolith in environments, the phytolith C sink rate can be estimated from data of PhytOC production rate and phytolith stability factor as:
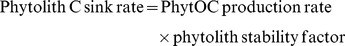
(3)where PhytOC production rate may be estimated from equation (2) and the phytolith stability factor is assumed to be 0.9±0.05 as most phytoliths have been proved stable for thousands of years though some small phytolith particles containing little carbon may be partly dissolved depending on formation sites and chemical composition of phytoliths in plant organs, and deposition environments of phytoliths after plant decay [18], [25].

## Results

### Distribution of PhytOC in Dominant Arable Crops

The global area of croplands is 1532.6 10^6^ hm^2^, about half of which is covered by cereals (Table 1). The PhytOC content varies greatly among different crops (0.02–0.25%, with an average of 0.13%) (Table 1). Generally, sugar cane and cereals have higher PhytOC contents in dry biomass (0.16–0.25%) than than other crops (0.02–0.08%). Within cereals, rice has higher PhytOC content in dry biomass (0.25±0.07%) than other cereal crops such as wheat (0.16±0.08%) and maize (0.16±0.05%).

**Table 1:** General information and PhytOC content of the dominant arable crops.

Farm crops	Area(10^6^ hm^2^)^a^	Plant Si-richorgans	PhytOC (%)^b^	
			mean	SE
Crops (total)	1532.6		0.13	0.05
Cereals (total)	697.7	Stem, sheath and leaf	0.19	0.07
Rice	164.1	Stem, sheath and leaf	0.25	0.07
Wheat	220.4	Stem, sheath and leaf	0.16	0.08
Maize	170.4	Stem, sheath and leaf	0.16	0.05
Soybeans	103	Stem and leaf	0.02	0.01
Roots and Tubers	54.3	Stem and leaf	0.02	0.01
Oil-bearing crops	62	Stem and leaf	0.08	0.08
Seed cotton	35.2	Stem and leaf	0.02	0.01
Sugar cane	25.4	Sheath and leaf	0.25	0.07

a) values from FAO (2012).

b) estimated from phytolith and silica content data (This study; ref. [19]–[21], [30]–[33]) using equation (1) and occluded-C content in phytolith of 3±1% (This study; ref. [19]–[21], [27]).

### Phytolith Carbon Sink of Global Croplands

The phytolith C sink varies greatly among different crops (Table 2). The phytolith C sinks generated by rice, wheat and maize (6.60±1.99, 4.93±2.30 and 6.14±2.46 Tg CO_2_ yr^−1^, respectively) are much higher than other crops. The total phytolith C sink produced by global farm crops is around 26.35±10.22 Tg CO_2_ yr^−1^, 85% of which is contributed from cereals, including rice (25%), wheat (19%) and maize (23%).

**Table 2:** Estimated phytolith C sink produced by global farm crops in 2011.

Farm crops	Crop output(Tg yr^−1^)^a^	Si-rich organfactor^b^	ANPP^c^ of Si-richorgans (Tg yr^−1^)^c^	Phytolith C sink (Tg CO_2_ yr^−1^)^d^	
				Mean	SE
Crops (total)		1.43	8091	26.35	10.22
Cereals (total)	2587	1.37	3557	22.39	8.41
Rice	723	1.1	795	6.60	1.99
Wheat	704	1.29	906	4.93	2.30
Maize	883	1.35	1194	6.14	2.46
Soybeans	261	1.5	391	0.27	0.14
Roots and Tubers	807	0.58	468	0.3	0.17
Oil-bearing crops	105	2.2	231	0.59	0.70
Seed cotton	77	2.91	225	0.18	0.07
Sugar cane^e^	1794	0.18	323	2.63	0.74

a values from FAO [29];

b mass ratios of the Si-rich organ: crop output from Huang et al. [35] and Zhu et al. [36];

c ANPP: above-ground net primary productivity;

d estimated from the crop output and Si-rich organ factor and PhytOC content in Table 1 using equations (2, 3);

e The crop output of sugar cane is fresh cane weight.

**Figure 2:**
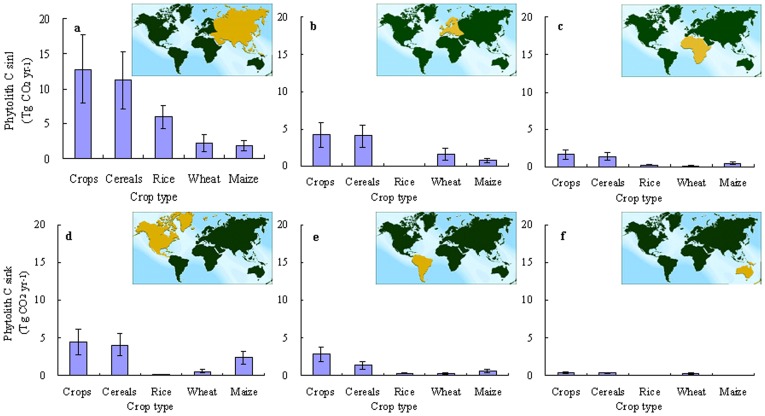
Phytolith carbon sink production by farm crops from different continents in 2011. Where **A:** Asia, **B:** Europe, **C:** Africa, **D:** North America, **E:** South America, **F:** Oceania. ‘Crops’ represents the sum of all farm crops and “Cereals” represents the sum of all cereal crops including rice, wheat, maize etc.

Figure 2 displays the relative land area of the major continents and the phytolith C sink produced by farm crops in each in 2011. The largest phytolith C sinks occur in Asia (12.80±4.90 Tg CO_2_ yr^−1^), North America (4.50±1.74 Tg CO_2_ yr^−1^) and Europe (4.21±1.66 Tg CO_2_ yr^−1^), which account for 49, 17 and 16% of the total global croplands, respectively.

**Figure 3:**
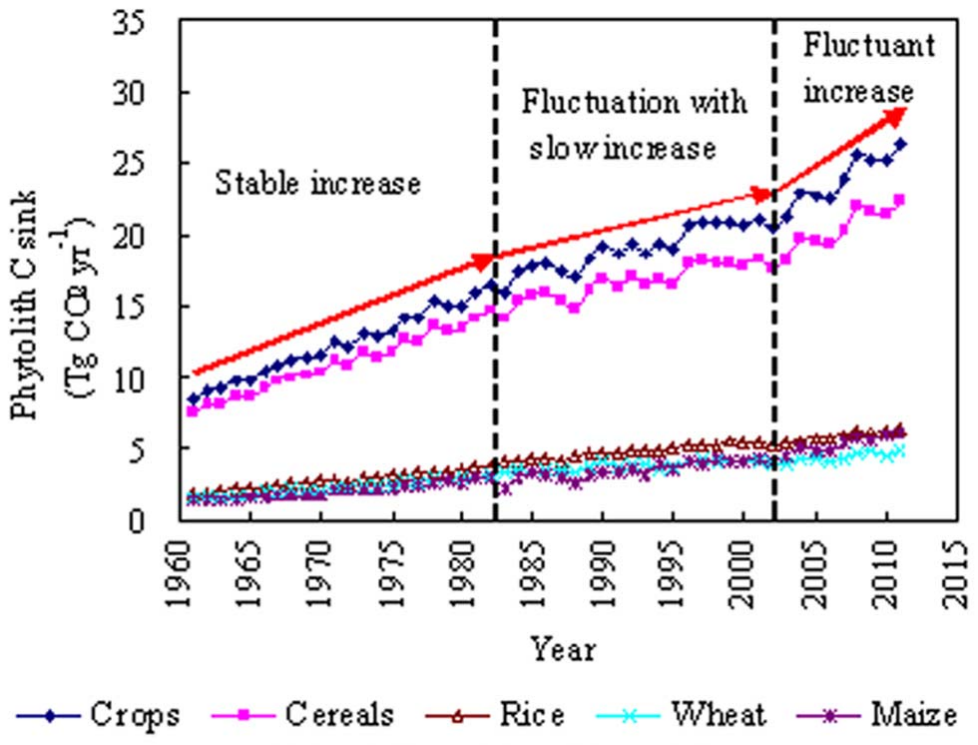
Phytolith carbon sink produced by global farm crops from 1961 to 2011. Crops include all farm crops in Table 1. Cereals include rice, wheat, maize, millet, barley and sorghum.

The total phytolith C sink of global croplands has tripled since 1961 (Figure 3). In general, the evolution of the phytolith C sink since 1961 may be divided into three stages:

1. 1961–1982: the total phytolith C sink increased steadily from 8.61 to 16.57 Tg CO_2_ yr^−1^.
2. 1983–2002: the total phytolith C sink fluctuated, with a slow increase from 15.99 to 20.51 Tg CO_2_ yr^−1^.
3. 2003–2011: the total phytolith C sink increased, with some fluctuation, from 21.14 to 26.35 Tg CO_2_ yr^−1^.

## Discussion

### Contribution of Phytolith C Sink to Global Cropland C Balance

By comparing phytolith C sink data and the global cropland soil C balance during 1961–2100, the contribution of the phytolith C sink to the net global cropland C balance was estimated (Table 3). Ruddiman [37] estimated that the emission from land-use conversion during the postindustrial era (i.e. 200 years), using 0.8 Gt C yr^–1^ (or 2.93 Pg CO_2_ yr^–1^), at 160 Gt C (or 587 Pg CO_2_). Using an average soil C sink rate of –2.93 Pg CO_2_ yr^–1^ during 1961–2015, the total soil C sink is about –161.2 Pg CO_2_. Lal [6], [14] estimated a high and attainable soil C sequestration potential of 0.55 Gt C yr^–1^ (or 2.02 Pg CO_2_ yr^–1^) for global croplands assuming judicious land use and recommended management practices (RMPs) were taken world-wide. Taking an average soil C sink rate of 2.02 Pg CO_2_ yr^–1^ during 2016–2100, the total soil C sink is about 171.7 Pg CO_2_. The total net soil C sink of global croplands during 1961–2100 is about 10.6 Pg CO_2_. Taking an average phytolith C sink rate of 0.03 Pg CO_2_ yr^–1^, the total phytolith C sink of global croplands during 1961 and 2100 is 4.2±1.9 Pg CO_2_ yr^–1^, 40±18% of the total net soil C sink (Table 3).

**Table 3:** Contribution of the phytolith C sink to the global cropland C balance for 1961–2100.

	Phytolith C	Soil C			Phytolith C contribution (%)
Period	1961–2100	1961–2015a)	2016–2100b)	1961–2100	1961–2100
Sink rate (Pg CO_2_ yr^−1^)	0.03±0.01	−2.93	2.02	0.08	40±18
Total sink (Pg CO_2_)	4.2±1.9	−161.2	171.7	10.6	40±18

Sinks are positive values and sources are negative values.

a) the average soil C sink rate data of 1961–2015 are after Ruddiman [37].

b) the average soil C sink rate data of 2016–2100 are after Lal [6], [14] assuming judicious land use and recommended management practices (RMPs) are applied worldwide during 2016–2100.

### Enhancing Phytolith Carbon Sink through the Optimization of Cropping System and Fertilization

Carbon sink trading has been carried out in many fields [38]. If phytolith carbon sink can be measured exactly, we believe that the sink may also be traded to increase the income of farmers. Therefore, in the future, farmers will be optimising carbon sequestration besides increasing yields.

Regional analysis of crop structures and farm productivity (FAO, 2012) suggests that the high phytolith C sinks in Asia, North America and Europe are due to the relatively wide production of rice, maize and wheat, respectively. The rapid increase of total phytolith C sink since 1961 has been due to cropland expansion and increase in the cereal yield per unit area as a result of fertilizer application and irrigation.

Although the global cropland area is difficult to increase significantly in the near future, the findings of the study suggest that the present global cropland phytolith carbon sink could be further enhanced through the optimization of cropping system and fertilization (Table 4).

**Table 4:** Potential measures to enhance global cropland phytolith carbon sink.

Types	Measures	Mechanisms	Comments
Optimization ofcropping system	Enhancement of cereal percentagein croplands	Enhancing crop output andphytolith content	High efficiency in all croplandswith low costs
	Enhancement of multi- cropping index	Enhancing crop output	High efficiency in all croplandswith low costs
Fertilization	Silicon fertilizer application	Enhancing crop phytolith content	High efficiency in cereal croplandsand sugarcane with high costs
	Rock powder amendment	Enhancing crop phytolith content	High efficiency in cereal croplandsand sugarcane with low costs
	Organic mulching	Enhancing crop output andphytolith content	High efficiency in cereal croplandsand sugarcane with low costs
	Traditional fertilization	Enhancing crop output	High efficiency with high costs

Cropping system optimization measures include enhancement of cereal area percentage in croplands and enhancement of multi-cropping index (Table 4). For example, Parr and Sullivan [20] and Li et al. [19] revealed that the enhancement of rice and wheat area percentage in croplands might significantly increase the total phytolith C sink in croplands because of their higher phytolith contents than other crops with low costs. Enhancement of multi-cropping index may significantly increase the total cropland phytolith C sink by enhancing crop output with low costs.

Fertilization measures include silicon fertilizer application, rock powder amendment, organic mulching, and traditional fertilization (Table 4). Silicon fertilizer application, rock powder amendment and organic mulching will increase soil bioavailable silicon input, plant silicon uptake and phytolith content for cereals and sugarcane [5], [19]. Traditional fertilization (N, P, K fertilizer application) may also increase total phytolith C sink in croplands by enhancing crop output.

Although the potential measures proposed for promoting cropland phytolith C sink based on the study are meritable, more data is required. The exact efficiency and costs of the proposed measures need further assessment before practical measures may be implemented to sequester globally-significant amounts of atmospheric CO_2_.

## Conclusions

Relative to the liable biomass C sink, the phytolith C sink in croplands is certain and stable, and can be sustained for several hundreds or thousands of years in most regions of the world. The phytolith sink of global croplands is a stable net sink of 26.35±10.22 Tg CO_2_ yr^−1^, and may play a significant role in global cropland C balance for 1961–2100. The high phytolith sinks in Asia, North America and Europe can be attributed to the relatively high production of rice, maize, and wheat, respectively. The total phytolith C sink of global croplands has tripled since 1961 mainly due to fertilization, irrigation and cropland expansion. Taking an average phytolith C sink rate of 0.03 Pg CO_2_ yr^–1^, the total phytolith C sink of global croplands during 1961 and 2100 is 4.2±1.9 Pg CO_2_ yr^–1^, 40±18% of the total net soil C sink. Our data suggest that the cropland phytolith C sinks may be further enhanced by adopting cropland management practices such as optimization of cropping system and fertilization.
